# A Manual of Refraction and Motility

**Published:** 1903-08

**Authors:** 


A Manual of Refraction and Motility. For Students and Practitioners of Medicine. By William Norwood Suter. M. D.. Assistant Surgeon to the Episcopal Eye, Ear and Throat Hospital, Washington, D. C. Duodecimo, 382 pages, with 101 engravings and four colored plates. Lea Brothers & Co., Philadelphia and New York. 1903. (Price; cloth, $2.00 net.)

Those wishing a condensed work on refraction and the motility of the eye, will find this a useful book for reference. Considerable space is devoted to the well-known mathematical formulæ illustrating the theory of refraction, some methods of determining refraction, together with short articles on hypermetropia, myopia, astigmatism, anisometropia and accommodation. Under disorders of motility a description of tests for the detection of the paralytic and nonparalytic are given; also, some of the best known operations for the relief of such conditions.

Combining refraction and motility in one book is an excellent idea, and the author is to be congratulated on making so complete a manual within 400 pages. R. H. S.

